# Multiple Interactions in Polar Lead‐Free Perovskites toward Highly Stable X‐Ray Detection

**DOI:** 10.1002/advs.202412504

**Published:** 2025-04-03

**Authors:** Chang Qu, Jianbo Wu, Zeng‐kui Zhu, Qianwen Guan, Huang Ye, Ruiqing Li, Chengshu Zhang, Yaru Geng, Hang Li, Lijun Xu, Haiqing Zhong, Ailin Wang, Chengmin Ji, Zhenyue Wu, Junhua Luo

**Affiliations:** ^1^ State Key Laboratory of Functional Crystals and Devices Fujian Institute of Research on the Structure of Matter Chinese Academy of Sciences Fuzhou Fujian 350002 P. R. China; ^2^ College of Chemistry and Materials Science Fujian Normal University Fuzhou 350007 P. R. China; ^3^ State Key Laboratory of Structural Chemistry, Fujian Institute of Research on the Structure of Matter Chinese Academy of Sciences Fuzhou Fujian 350002 China; ^4^ Fujian College University of Chinese Academy of Sciences Fuzhou Fujian 350002 China

**Keywords:** interactions, ion migration, operational stability, polar lead‐free perovskites, self‐driven X‐ray detection

## Abstract

Lead‐free halide perovskites have emerged as a promising class of high‐performance “green” X‐ray detecting semiconductors due to their nontoxicity and strong X‐ray absorption. However, ion migration caused by high operating electric field remains a bottleneck limiting the long‐term stability of perovskite X‐ray detectors. Herein, by introducing multiple halogen interactions in lead‐free perovskites, stable X‐ray detection is successfully realized. Specifically, 0D polar bismuth halide perovskites (*R*/*S*‐BPEA)_4_Bi_2_I_10_ (**1*R***/**1*S***, *R*/*S*‐BPEA = *R*/*S*‐1‐(4‐bromophenyl)ethylammonium) are designed by introducing Br‐substituted chiral organic cation BPEA, which exists with the molecular electrostatic forces between the Br atom and neighboring benzene ring and halogen interaction of Br···I. Notably, their introduction improves the activation energy of ion migration, which makes the dark current drift of the X‐ray detector as low as 3.25 × 10^−8^ nA cm^−1^ s^−1^ V^−1^ at 2500 V cm^−1^. Furthermore, the excellent operational stability under prolonged X‐ray irradiation and unchanged device sensitivity after 90 days of exposure to air, further demonstrates the improved stability of perovskites. Meanwhile, the chiral‐polar characteristic of the **1*R***/**1*S*** gives them potential for self‐powered detection, with a low detection limit of 183 nGy s^−1^ at zero bias for single‐crystal devices. This study opens new avenues for the future development of “green”, highly stable, self‐powered radiation detectors.

## Graphical abstract

In this work, 0D polar lead‐free halide perovskites (*R*/*S*‐BPEA)_4_Bi_2_I_10_ (*R*/*S*‐BPEA = *R*/*S*‐1‐(4‐bromophenyl)ethylammonium) with multiple interactions are successfully constructed by introducing a chiral organic cation BPEA. The devices based on their high‐quality SC exhibit excellent self‐driven X‐ray detection performance and high operational stability over long periods of exposure to X‐ray irradiation at high operating electric fields.

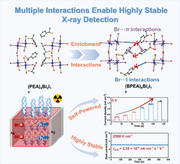

## 1 Introduction

Direct X‐ray detectors, which can directly convert X‐rays into electrical signals, are widely applied in security screening, medical imaging, non‐destructive manufacturing inspection, scientific research, etc.^[^ ^1^, ^2^, ^3^, ^4^, ^5^, ^6^, ^7^, ^8^ ^]^ However, conventional X‐ray detectors such as *α*‐Se,^[^ ^9^ ^]^ Si,^[^ ^10^ ^]^ and Cd(Zn)Te^[^ ^11^, ^12^ ^]^ have the disadvantages of high preparation cost and small absorption coefficients for X‐rays. Therefore, the investigation of novel materials for the development of high‐performance X‐ray detectors has emerged as a research focus within the field of X‐ray detection. Recently, organic‐inorganic hybrid perovskites (OIHPs) have gained significant progress in direct X‐ray detection owing to their significant X‐ray absorption coefficients, high mobility lifetime (*µτ*) product, low trap density, and low‐cost preparation.^[^ ^1^, ^13^, ^14^, ^15^, ^16^ ^]^ E.g., He et al. reported a direct lead halide perovskite computed tomography (CT) imager with an ultra‐low total X‐ray radiation dose rate via a low‐cost coating process, achieving a breakthrough in the field of perovskite CT.^[^ ^8^ ^]^ In addition, the X‐ray detector based on 3D MAPbI_3_ with an ultra‐low detection limit of 0.1 nGy s^−1^ and a high sensitivity of 5.2 × 10^6^ µC Gy^−1^ cm^−2^ has been assembled, which is considerably higher than that of conventional X‐ray detectors.^[^ ^17^ ^]^ Despite the significant success of 3D OIHPs, most of them suffer from severe ion migration, resulting in large dark current and severe dark current drift under highly applied electric fields. Additionally, the migrating ions may corrode the metal electrodes, which impacts the long‐term operational stability of the devices, severely limiting their practical application in X‐ray detectors.^[^ ^18^, ^19^, ^20^, ^21^ ^]^

Comparatively, low‐dimensional perovskites have demonstrated significant promise in inhibiting ion migration, thereby ensuring stable X‐ray detection.^[^ ^22^, ^23^, ^24^ ^]^ For example, the 2D (4‐ABA)PbI_4_ (4‐ABA = 4‐aminobenzylamine) perovskite detector exhibits a small dark current drift of 5.18 × 10^−8^ nA cm^−1^ s^−1^ V^−1^ at 10 V bias.^[^ ^25^ ^]^ In order to further suppress ion migration and improve device stability, the researchers proposed a strategy of introducing halogens into low‐dimensional OIHPs to enhance the halogen interactions within the lattice.^[^ ^26^, ^27^, ^28^, ^29^ ^]^ For instance, Fu and colleagues successfully inhibited the ion migration in a 2D lead halide perovskite of (I‐MBA)_2_PbI_4_ (I‐MBA = 1‐(4‐iodophenyl)ethylamine) by introducing strong and homogeneous halogen bonds between the layers via an interlayer‐locked structure, thereby obtaining a stable pure red perovskite light‐emitting diodes.^[^ ^30^ ^]^ Zhang et al. synthesized (o‐F‐PEA)_2_PbI_4_ (F‐PEA^+^ = fluorophenylethylammonium) perovskite single crystals (SCs) by introducing fluorine atoms at the orthogonal position of phenylethylamine, which inhibits ionic migration by using electrostatic interactions between the fluorine and benzene ring, which results in a substantial improvement in the photocurrent stability of X‐ray detector.^[^ ^31^ ^]^ Nevertheless, almost all the core components of these low‐dimensional perovskites using this strategy contain toxic lead (Pb), which presents a significant threat to human health and the environment and hinders their further production. Therefore, there is an urgent need to explore non‐toxic and stable X‐ray detectors.^[^ ^22^, ^32^, ^33^, ^34^, ^35^ ^]^ Recently, bismuth‐based perovskites have shown significant advantages in the area of “green” X‐ray detection, owing to their non‐toxic nature and impressive ability to absorb X‐rays,^[^ ^36^, ^37^, ^38^ ^]^ such as a self‐powered X‐ray detector based on (*R*/*S*‐PPA)_2_BiI_5_ (*R*/*S*‐PPA = *R*/*S*‐1‐phenylpropylamine) with a detection limit of 270 nGy s^−1^,^[^ ^39^ ^]^ and the AG_3_Bi_2_I_9_ (AG = aminoguanidinium) SC X‐ray detectors exhibit a remarkable sensitivity of 5791 µC Gy^−1^ cm^−2^ at 50 V bias,^[^ ^40^ ^]^ etc. However, the stability of the X‐ray detectors fabricated on the bismuth‐based perovskites SC is still a pressing issue at high bias. Inspired by this, it is a useful strategy to achieve stable and “green” X‐ray detection in low‐dimensional bismuth halide perovskite crystals (BHPs) by introducing halogen interactions to suppress ion migration.

Herein, we design and synthesize 0D BHPs (*R*/*S*‐BPEA)_4_Bi_2_I_10_ (**1*R*/1*S***, *R*/*S*‐BPEA = *R*/*S*‐1‐(4‐bromophenyl)ethylammonium) SCs with multiple intermolecular electrostatic forces by introducing the halogen‐substituted chiral organic cation *R*/*S*‐BPEA. Notably, compared to 0D dinuclear polar BHPs (*R*/*S*‐PEA)_4_Bi_2_I_10_ (**2*R***/**2*S***), the presence of Br···I as well as Br···π interactions in **1*R***/**1*S*** improves the ion migration energy and blocks the ion migration paths, resulting in more stable X‐ray detection. Specifically, the devices based on **1*S*** high‐quality SCs exhibit a low dark current drift of 3.25 × 10^−8^ nA cm^−1^ s^−1^ at a high operating electric field of 2500 V cm^−1^, excellent operational stability under continuous prolonged irradiation of X‐ray, and the almost unchanged device sensitivity after 90 days of exposure to air. In addition, the devices have a low detection limit in self‐driven mode (183 nGy s^−1^). Our work has successfully designed chiral polar BHPs with multiple interactions and achieved self‐powered highly stable X‐ray detection, which will provide new ideas for constructing environmentally friendly self‐powered X‐ray detectors in the future.

## 2 Result and Discussion

### 2.1 Crystal Structure and Characterization

**Figure 1:**
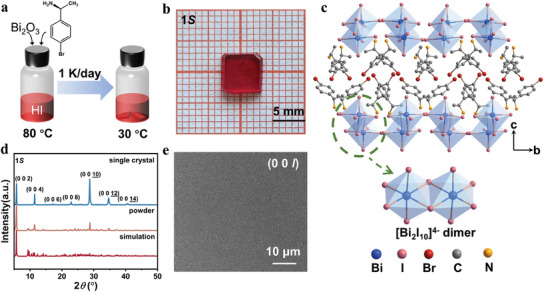
a) Schematic of crystals growth. b) Photograph of the grown high‐quality crystal of **1*S***. c) Packed crystal structure of **1*S*** along the *a*‐axis. d) PXRD patterns of powder and bulk **1*S*** SC. e) SEM image of (00*l*) crystal surface of **1*S*** SC.

High‐quality red bulk SCs of **1*S*** with dimensions of 6 × 6 × 1 mm^3^ were synthesized by stoichiometric reaction in a hot hydriodic acid solution containing Bi_2_O_3_ and *R*/*S*‐BPEA using a slow temperature‐cooling approach (**Figure** **1**a,b). According to Table S1 (Supporting Information), **1*R*** and **1*S*** belong to the monoclinic crystal system with chiral‐polar space group *P*2_1_, as determined by single‐crystal X‐ray diffraction (SCXRD). Considering the chiral‐polar structures of **1*R*** and **1*S***, their chiral properties were investigated using circular dichroism (CD) spectroscopy. Figure S2 (Supporting Information) shows that distinct CD signals are observed at almost the same wavelengths for **1*R*** and **1*S*** with opposite sign values.^[^ ^41^ ^]^ According to the point‐charge model, the spontaneous polarization (*P*_s_) along the *b*‐axis was subsequently calculated to be 0.366 µC cm^−2^ (Figure S4 and Table S2, Supporting Information). Notably, the intrinsic polar structure of **1*R*/1*S*** generates an internal electric field that enables self‐powered detection. As depicted in Figure 1c, the chiral *R*/*S*‐BPEA organic cations distribute around the isolated [Bi_2_I_10_]^4‐^ dimer with two edge‐sharing [BiI_6_]^3−^ octahedra, forming a typical 0D dinuclear structure. Moreover, the chiral cations are anchored to the inorganic framework [Bi_2_I_10_]^4‐^ via N‐H··I hydrogen bonds. The high phase purity of **1*S*** is confirmed by powder X‐ray diffraction (PXRD), in which the measured and simulated diffraction PXRD patterns are in good match (Figure 1d). XRD analysis is conducted on a bulk crystal, which exhibits well‐oriented peaks corresponding to the (00*l*) plane (*l* = 2, 4, 6, 8, 10, 12, 14), suggesting its well‐oriented single‐crystalline lattice and preferential growth. The crystal surface appears smooth (00*l*) according to the scanning electron microscopy (SEM) and atomic force microscopy (AFM) images (Figure 1e; Figure S5, Supporting Information), demonstrating the excellent crystal quality of **1*S*** SCs. As can be seen by observing the X‐ray energy dispersive spectroscopy (EDS) mapping of **1** (Figure S6, Supporting Information), the elements Bi, I, Br, C, and N are uniformly distributed in the crystal. In addition, we prepared **2*R***/**2*S*** (CCDC 2 193 356, 2 193 357) with the same structural features as **1*R***/**1*S*** by the same methods, and the phase purity was verified by PXRD (Figure S8, Supporting Information).^[^ ^42^ ^]^ Overall, we have successfully synthesized high‐quality SCs with intrinsic polar structures, providing potential candidates for excellent performance X‐ray detection.

### 2.2 Molecular Interactions

**Figure 2:**
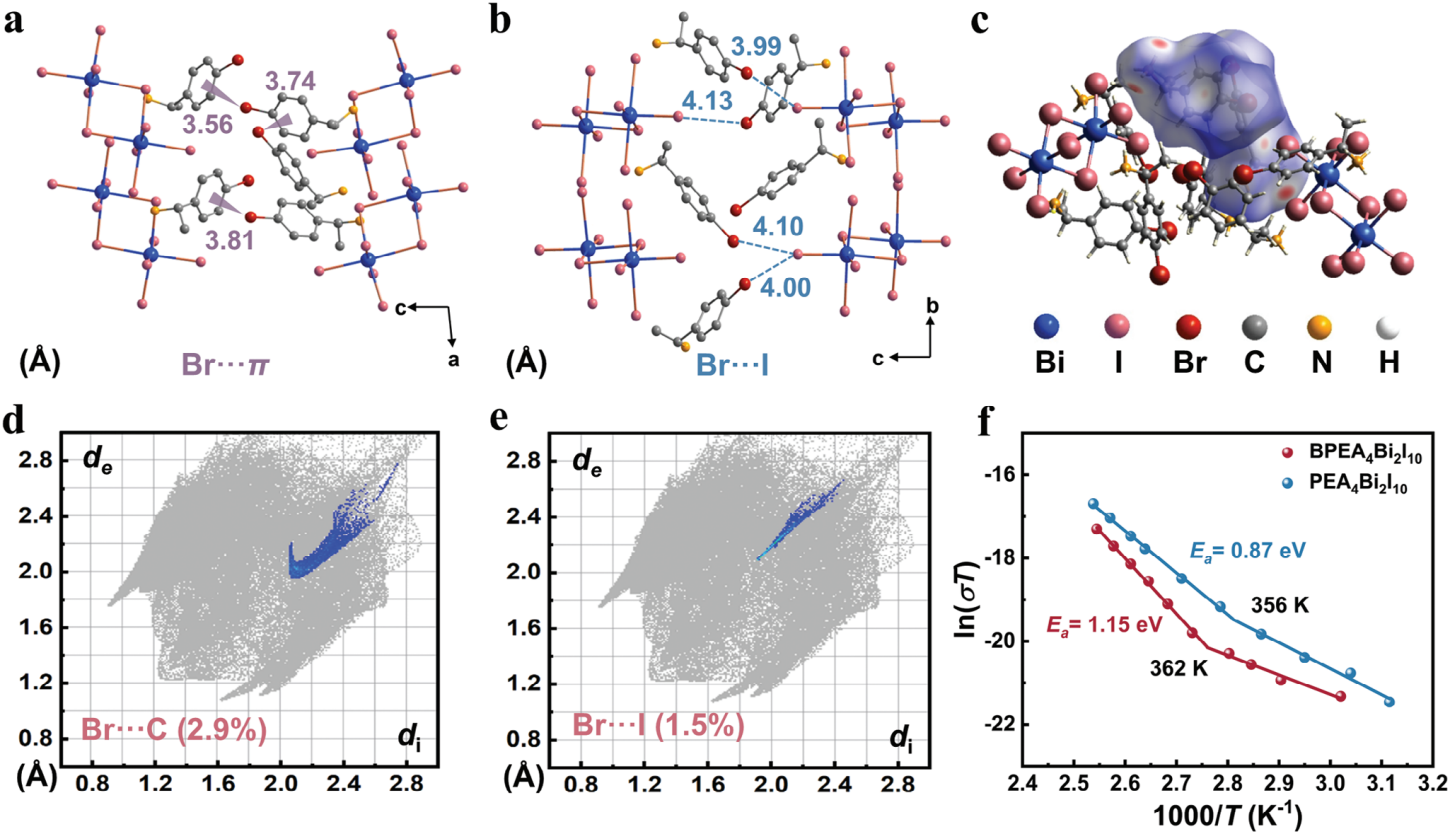
a) Distances from Br atoms to neighboring benzene rings in the structure of **1*S***. b) Distances from Br atoms to neighboring I atoms in the structure of **1*S***. c–e) Hirshfeld surface analyses of the BPEA cations and their corresponding 2D fingerprints of Br⋯π and Br⋯I contacts. f) Temperature‐dependent conductivity of **1*S*** and **2*S*** SCs.

To reveal the role of the bromine‐substituted aromatic cation BPEA in 0D BHPs, we analyzed a variety of molecular interactions in **1*S***. As depicted in **Figure** **2**a, the shortest distance between the Br atom on the aromatic cation BPEA and the neighboring benzene ring is 3.56 Å.^[^ ^33^, ^43^ ^]^ The shorter distance can be speculated to generate additional molecular electrostatic forces, which strengthens the connection between organic cations. In addition, there are halogen‐halogen interactions (Br⋯I) between the Br atom within the organic cation and the I atom on the inorganic framework [Bi_2_I_10_]^4‐^ dimer, with a shortest distance of 3.99 Å (Figure 2b). At the same time, Br⋯Br interactions were also present between neighboring BPEA cations (Figure S9, Supporting Information). Moreover, we performed Hirschfeld surface analysis and 2D fingerprinting of **1*S*** using Crystal Explorer software to demonstrate intramolecular non‐covalent interactions.^[^ ^44^ ^]^ The Br atom exhibits strong electrostatic interactions with the benzene ring (Br···π) as demonstrated in Figure 2c–e and Figure S10 (Supporting Information), which is consistent with the above results. Furthermore, thermogravimetric analysis measurements were performed on samples of **2*S*** and **1*S*** SCs. As shown in Figure S11 (Supporting Information), the decomposition temperature of **1*S*** SC is 496K higher than **2*S*** (486K) without bromine substitution. Halogen‐halogen interactions and halogen⋯π interactions in halide perovskites are beneficial for enhancing structural stability and inhibiting ion migration, thus facilitating stable operational detection.

To gain more insight into the ion migration mechanism of this SC, the activation energy (*E*_a_) of ion migration was deduced from the temperature‐dependent conductivity curves according to the Nernst‐Einstein equation,

(1) $$\ln\left(\sigma T\right)=\ln\sigma_{0}-E_{a}\left(1/k_{B}T\right)$$

where *σ*_0_ is a constant, *k*_B_ and *T* are Boltzmann's constant (8.617 × 10^−5^ eV K^−1^) and temperature, respectively. As the temperature rises, the conductivity of perovskite SC undergoes transitions from electronic to ionic. For **1*S*** SC with multiple interactions, the ionic conductivity dominates when the temperature is increased to 362 K, giving an *E*_a_ of 1.15 eV based on the slope of the high‐temperature region (Figure 2f). This value is not only much higher than that of the 0D BHPs, FA_3_Bi_2_I_9_ (357 K, *E*_a_ = 0.56 eV),^[^ ^45^ ^]^ but also exceeds many 2D halide perovskites, such as (NH_4_)_3_Bi_2_I_9_ (350 K, *E*_a_ = 0.91 eV),^[^ ^22^ ^]^ I‐MBA_2_PbI_4_ (315 K, *E*_a_ = 0.91 eV).^[^ ^30^ ^]^ In contrast, the transition temperature of **2*S***, which has no halogen on the organic cation, decreases to 356 K and *E*_a_ drops to 0.87 eV. Such a result suggests that the strong interactions within the perovskite lattice effectively increase the energy barrier for ion migration, ultimately inhibiting ion migration. Therefore, **1*S*** is expected to realize a more stable X‐ray detection.

### 2.3 X‐Ray Detection Performance

**Figure 3:**
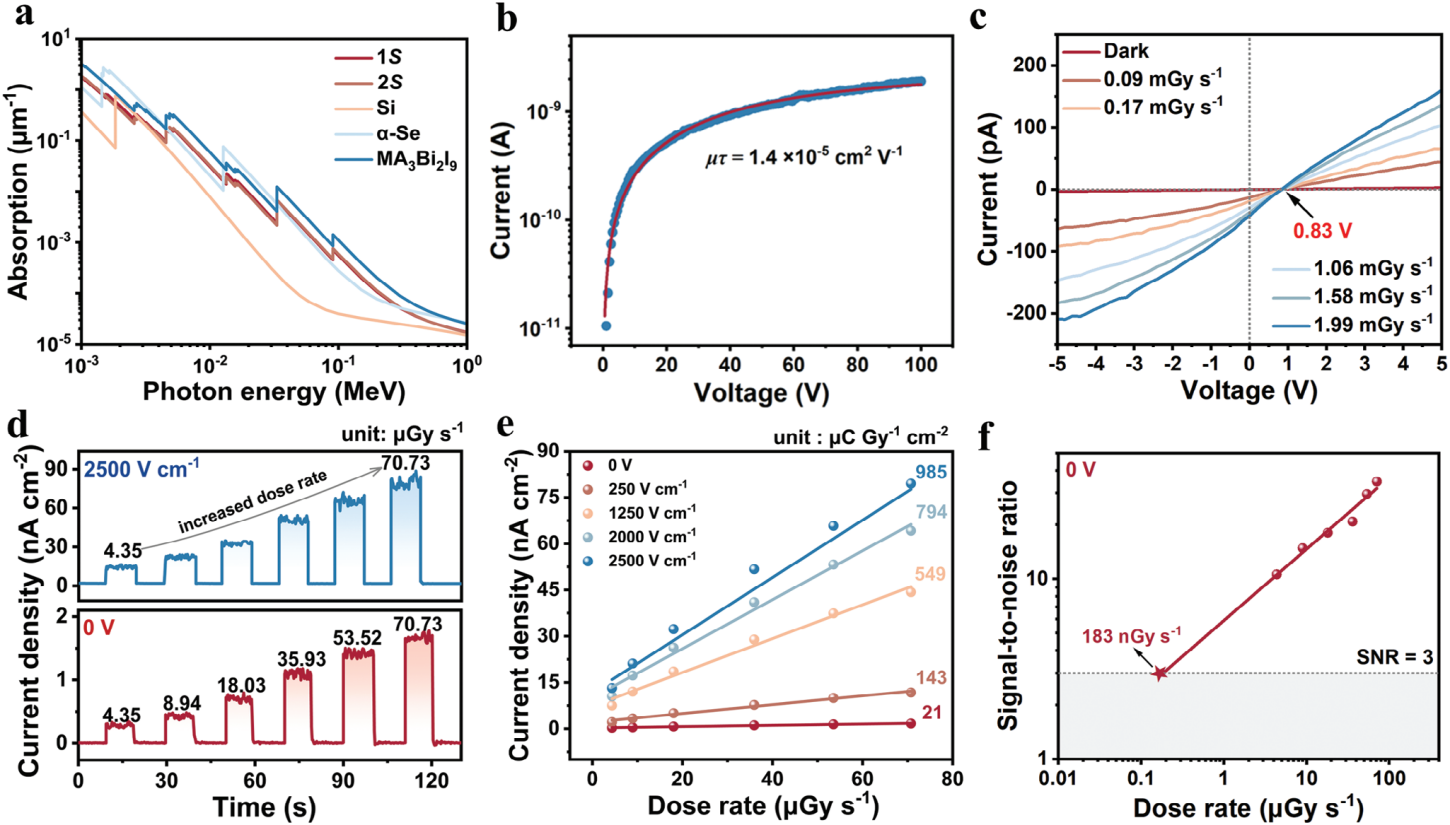
a) X‐ray absorption spectra of **1*S***, **2*S***, Si, 𝛼‐Se and MA_3_Bi_2_I_9_. b) Voltage‐dependent X‐ray conductivity of the **1*S*** SC device. c) *I*‐*V* curves of **1*S*** devices under X‐ray radiation. d) *J‐t* curves of **1*S*** SC detector under increased X‐ray dose rates at 0 V and 2500 V cm^−1^, respectively. e) The photocurrent densities as a function of dose rate under different electric fields. The slope of the fitted line indicates the sensitivity. f) The detection limit of **1*S*** at 0 V bias.

To evaluate the semiconducting properties of **1*S***, we first determined the optical bandgaps of **1*S*** using ultraviolet‐visible absorption spectroscopy. As shown in Figure S12 (Supporting Information), a clear absorption cutoff edge of **1*S*** is observed near ≈627 nm, and the optical bandgap is further determined by fitting Tauc's equation, which is estimated to be 2.00 eV. This value is comparable to the bandgap of other reported 0D binuclear chiral BHPs such as (*R*/*S*‐1‐phenylpropylamine)_2_BiI_5_ (2.17 eV)^[^ ^39^ ^]^ and (*R*‐1‐(4‐fluoro)phenylethyl‐ammonium)_4_Bi_2_I_10_ (2.09 eV).^[^ ^46^ ^]^ The electronic structure of **1*S*** has been calculated by first‐principles density‐functional theory (DFT). Figure S13 (Supporting Information) shows that the bandgap value is 2.099 eV, which is comparable to the experimental value with the indirect bandgap feature. Further analysis of the partial density of states (PDOS) of **1*S*** reveals that the I‐5*p* state mainly contributes to the valence band maximum (VBM), whereas the conduction band minimum (CBM) is from the Bi‐6*p* orbital (Figure S14, Supporting Information). This result suggests that the inorganic framework of **1*S*** plays a crucial role in determining the semiconductor properties. Furthermore, we measured the current density‐voltage curve of **1*S*** SC, yielding a bulk resistivity (𝜌) of 2.5 × 10^11^ Ω cm (Figure S15, Supporting Information), which exceeds many 0D binuclear BHPs, such as AG_3_Bi_2_I_9_ (3.78 × 10^10^ Ω cm),^[^ ^40^ ^]^ Cs_3_Bi_2_I_9_ (2.79 × 10^10^ Ω cm),^[^ ^47^ ^]^ and it is ≈7.6 times larger than that of the **2*S*** (3.3 × 10^10^ Ω cm) (Figure S16, Supporting Information). Large resistivity is crucial for high‐performance X‐ray detectors, because it suppresses current noise and dark current.^[^ ^48^ ^]^ Then, according to the relation *α* ∝ *Z* ^4^ /*E* ^3^ (*E* is the X‐ray photon energy and *α* is the X‐ray attenuation coefficient), the **1*S*** structure is known to exhibit a large X‐ray absorption coefficient due to the high atomic number of elements Bi (*Z* = 83) and I (*Z* = 53). To quantify the X‐ray absorption, we first calculated the absorption spectra of **1*S*** and **2*S*** using photon cross‐section database simulations.^[^ ^49^ ^]^ **1*S*** and **2*S*** exhibit significantly higher X‐ray absorption coefficients than silicon (Si) and commercial *α*‐Se in a broad range of photon energies, as depicted in **Figure** **3**a. Higher absorption coefficients are associated with higher attenuation coefficients. The attenuation efficiency of these materials as a function of thickness at an X‐ray photon energy of 50 keV is displayed in Figure S17 (Supporting Information). To achieve full photon absorption, the high attenuation efficiency permits a reduction in crystal thickness. At the same thickness of 1 mm, the attenuation of incident X‐ray photons by **1*S*** is 89.1%, compared to 8.7% for Si and 83.0% for *α*‐Se, which indicates that **1*S*** has excellent X‐ray attenuation ability and great potential for photogenerated carrier production under irradiation of X‐ray. High‐performance devices often require efficient charge collection, which corresponds to having a high *µτ*. Figure S18 (Supporting Information) shows an Ag/**1*S*** SC/Ag two‐terminal structure device fabricated using a high‐quality **1*S*** SC with the electrode orientation along a direction parallel to the polar axis. Under continuous irradiation with 50 keV high‐energy X‐rays, we used the modified Hecht equation to fit the voltage‐dependent photoconductivity of **1*S*** to obtain a **1*S*** device‐based *µτ* product,^[^ ^1^ ^]^

(2) $$I=\frac{I_{o}\mu \tau V}{L^{2}}\left[1-\exp\left(\frac{-L^{2}}{\mu \tau V}\right)\right]$$

where *I* and *I_0_* are the photocurrent and the saturation photocurrent, *L* is the electrode spacing, *V* is bias voltage, *µ* and *τ* are the carrier mobility and carrier lifetime, respectively. The *µτ* product of the **1*S*** SC is 1.4 × 10^−5^ cm^2^ V^−1^ (Figure 3b), which is comparable to those of some lead‐free perovskite X‐ray detectors such as (*R*‐PPA)_2_BiI_5_ (5.6 × 10^−5^ cm^2^ V^−1^)^[^ ^39^ ^]^ and (*R*‐MPA)_4_AgBiI_8_ (*R*‐MPA = *R*‐*β*‐methylphenethylammonium) (2.2 × 10^−5^ cm^2^ V^−1^),^[^ ^50^ ^]^ etc. Strong X‐ray absorption and high *µτ* product reveal that **1*S*** has excellent potential for X‐ray detection. Figure 3c shows typical current‐voltage (*I*‐*V*) curves of the device measured under dark and X‐ray irradiation at different dose rates. The device exhibits an open‐circuit photovoltage (*V_oc_* = 0.83 V) under X‐ray irradiation, indicating a bulk photovoltaic effect (BPVE) of **1*S*** SC along the *b*‐axis direction. The intrinsic BPVE shows that the SC device can independently facilitate the separation and transport of photo‐generated carriers without external bias, demonstrating that **1*S*** can achieve self‐driven X‐ray detection. The photocurrent density of the **1*S*** SC increases linearly with the X‐ray dose rate from 4.35 µGy s^−1^ to 70.73 µGy s^−1^ at 0 V bias. Meanwhile, **1*S*** shows a rapid response with 161 ms (rise) and 226 ms (decay) (Figure S20, Supporting Information).Two critical metrics are utilized to evaluate the quantitative performance of detectors: sensitivity and detection limit. Sensitivity is defined by the amount of charge collected per unit area after exposure to radiation, which can be calculated using the formula,^[^ ^48^ ^]^

(3) $$S=\left(J_{ph}-J_{d}\right)/D$$

where *S* is the sensitivity, *D* is the X‐ray dose rate, *J_ph_* is the photocurrent density under X‐ray irradiation, *J_d_* is the current density under dark conditions. Figure 3e shows the linear correlation between the photocurrent density and the X‐ray dose rate. The sensitivity of **1*S*** can be obtained by fitting the slope of its *J*‐*D* curve to 21 µC Gy^−1^ cm^−2^ under 0 V bias, indicating a great self‐powered detection. The detection limit of an X‐ray detector represents the lowest X‐ray dose rate used for inspection, which is crucial for medical diagnostics and non‐destructive testing. As defined by the International Union of Pure and Applied Chemistry (IUPAC), the detection limit is the dose rate corresponding to a signal‐to‐noise ratio (SNR) of three. The SNR can be calculated as follows:

(4) $$SNR=\frac{{\bar{I}}_{photo}-{\bar{I}}_{dark}}{\sqrt{\frac{1}{N}\sum_{i}^{n}{\left(I_{i}-{\bar{I}}_{photo}\right)}^{2}}}$$

where the ${\bar{I}}_{\mathrm{photo}}$ and ${\bar{I}}_{\mathrm{dark}}$ are the average photocurrent and the average dark current, respectively, and the *I*_i_ denotes the measured photocurrent. As shown in Figure 3f, the SNR of the **1*S*** SC device is determined to be as high as 10.61 under a low dose rate of 4.35 µGy s^−1^ at 0 V bias. The dose‐rate‐dependent SNR fitting line has been extended to 3, and a detection limit as low as 183 nGy s^−1^ for the **1*S*** SC detector in self‐driven mode, which is almost 30 times lower than that of conventional medical diagnostic requirements (5.5 µGy s^−1^).^[^ ^5^ ^]^ As a result, **1*S*** SC devices are beneficial in reducing the risk of X‐ray irradiation. For comparison, we also performed X‐ray response tests at different electric field strengths (i.e., 250, 1250, 2000, and 2500 V cm^−1^) and different X‐ray dose rates (Figure 3d; Figures S21–24, Supporting Information). The *J*_ph_ is significantly greater (82.04 nA cm^−2^) than that at zero bias (1.78 nA cm^−2^) when exposed to the 70.73 µGy s^−1^ at 2500 V cm^−1^, demonstrating that high external electric field promotes the charge collection. However, the ion migration leads to unstable device operation at high electric field such as severe dark current shift. Notably, the response and baseline of the **1*S*** SC are stable at a large operating external electric field of 2500 V cm^−1^ and exhibit excellent operational stability, due to multiple interactions in **1*S***. In addition, as the electric field increases, the sensitivity of the detector also increases gradually. With a 2500 V cm^−1^, the detector can reach a high sensitivity of 985 µC Gy^−1^ cm^−2^, which is far higher than that of commercial *α*‐Se (20 µC Gy^−1^ cm^−2^, 10 V µm^−1^).^[^ ^9^ ^]^

**Figure 4:**
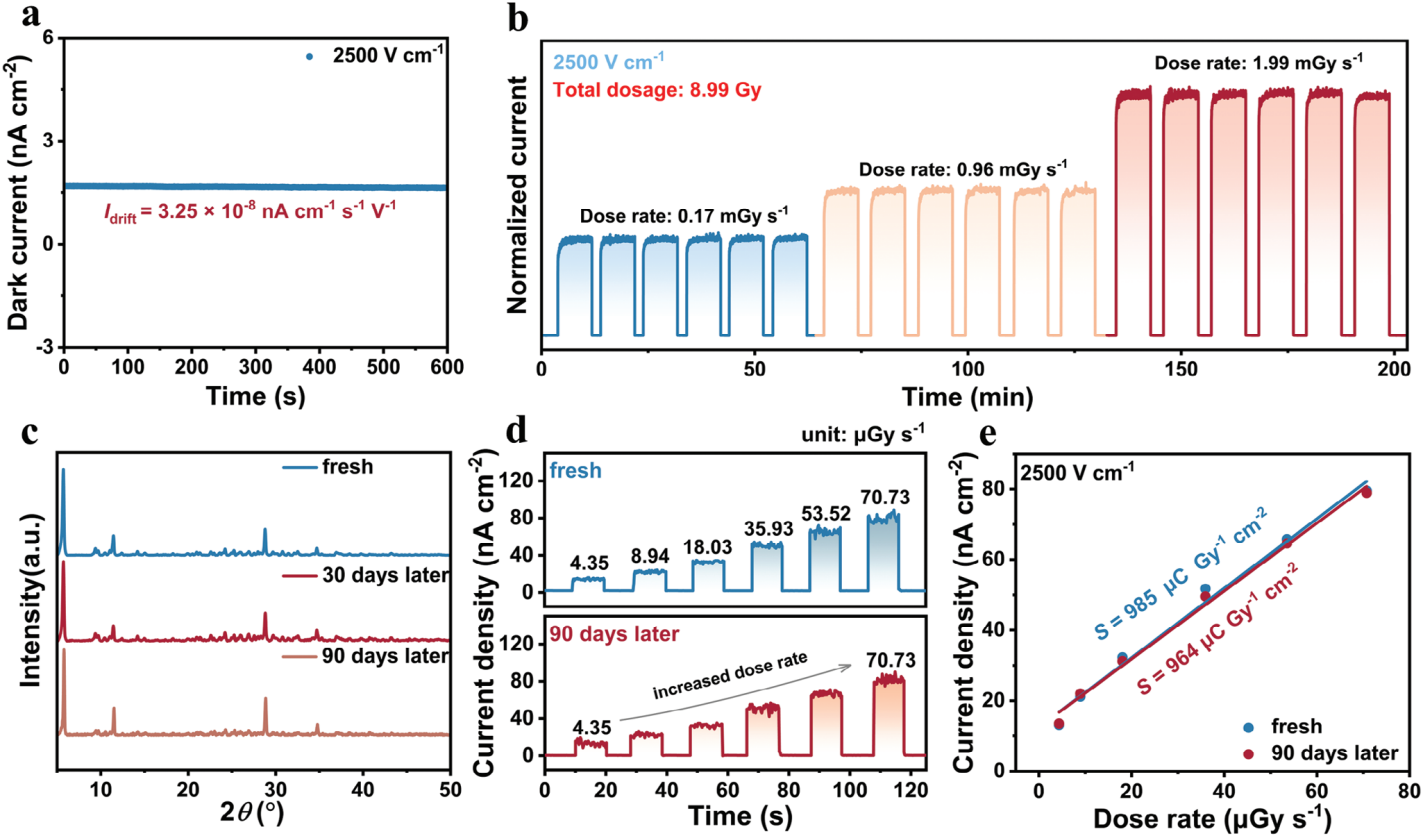
a) Dark current tracking of **1*S*** detector at 2500 V cm^−1^. b) Stabilized photocurrent of **1*S*** devices under long‐term X‐ray irradiation at 2500 V cm^−1^. The X‐ray total dosage is 8.99 Gy. c) PXRD patterns of **1*S*** during the freshness and following 90 days of exposure to the air. d) Stability measurement of the device under ambient conditions (RH 60% and 25 °C) for 90 days at 2500 V cm^−1^. e) Comparison of sensitivity in the fresh state and after 90 days of aging with applied electric field 2500 V cm^−1^.

Moreover, to evaluate the equipment stability of **1*S***, we introduce dark current drift (*I*_drift_) to quantify the dark current stability. The *I*_drift_ of an X‐ray detector is another crucial parameter, which can be calculated according to the following equation,^[^ ^51^ ^]^

(5) $$I_{drift}=\left(I_{t}-I_{0}\right)/\left(E\times A\times t\right)$$

where *I_t_* is the current at the moment, *I_0_ is* the initial current, *E* and *A* are the electric field and device area, respectively. **Figure** **4**a clearly demonstrates that the **1*S*** device exhibits an ultralow *I*_drift_ of 3.25 × 10^−8^ nA cm^−1^ s^−1^ V^−1^ at 2500 V cm^−1^. Notably, the *I*_drift_ of **1*S*** SC devices remains stable under large applied electric fields and is much lower than some previously reported lead‐free perovskites, such as (*R*‐PPA)_2_BiI_5_ SC (1.40 × 10^−4^ nA cm^−1^ s^−1^ V ^−1^, 10 V),^[^ ^39^ ^]^ and Rb_3_Bi_2_I_9_ (1.82 × 10^−7^ nA cm^−1^ s^−1^ V^−1^, 100 V).^[^ ^52^ ^]^ Meanwhile, the *I*_drift_ of **1*S*** SC devices is three orders of magnitude smaller than the **2*S*** at 250 Vcm^−1^ (Figure S29, Supporting Information). This suggests that the multiple interactions (i.e., Br⋯π, Br⋯Br, and Br⋯I) present in **1*S*** SCs can effectively inhibit ion migration and stabilize their dark currents. Further, continuous X‐ray irradiation was applied to the **1*S*** SC‐based detector to assess its operational stability at an external electric field of 2500 V cm^−1^. It was observed that the baseline and photocurrent of the **1*S*** SC detector exhibited high stability and reproducibility under different doses of continuous X‐ray irradiation (Figure 4b). Notably, neither photocurrent nor dark current remained changed at a total dose of 8.99 Gy, highlighting the irradiation stability of the **1*S*** SC detector in practical applications. Finally, we performed the environmental stability of **1*S*** and **1*S*** SC devices. Following a 90 days period of exposure to ambient air (RH 60% and 25 °C), **1*S*** crystal powders showed no significant change in the diffraction peaks in the PXRD pattern (Figure 4c), verifying its high phase stability. Meanwhile, we investigated the stability and repeatability of the **1*S*** SC detector in diverse environmental contexts and key indicators for evaluating their usability. Our long‐term follow‐up tests on unpacked **1*S*** SC detectors found no significant change in response current after 90 days (Figure 4d), and the sensitivity remained almost unchanged under the 2500 V cm^−1^ high external electric field (Figure 4e). The operational and environmental stability of the **1*S*** SC device demonstrates that **1*S*** SC inhibits ion migration due to the presence of multiple interactions, highlighting its potential for use in commercial applications.

## 3 Conclusion

In conclusion, we have successfully achieved a highly stable X‐ray detector by constructing 0D chiral polar BHPs (*R*/*S*‐BPEA)_4_Bi_2_I_10_ (**1*R***/**1*S***). The experimental results show that **1*S*** has a higher bulk resistivity and a larger mobility lifetime (*µτ*) product due to the Br‐substituted organic spacer in the crystal structure of **1*S***, which synergistically suppresses the ion migration path and further achieves a more stable X‐ray detection through the formation of strong interactions between Br atom and neighboring benzene ring, as well as with the I atoms on the inorganic perovskite framework. Besides, the X‐ray detector based on **1*S*** SCs has a sensitivity of up to 985 µC Gy^−1^ cm^−2^, and a low dark current drift of 3.25 × 10^−8^ nA cm^−1^ s^−1^ V^−1^ at a high external electric field of 2500 V cm^−1^. More interestingly, it shows high operational stability even after prolonged exposure to X‐ray irradiation at high electric fields. Meanwhile, after 90 days of exposure to air, the sensitivity of the device remained almost unchanged. This study introduces the concept of interactions in lead‐free polar semiconductors, and this work provides new ideas for the future development of environmentally friendly, highly stable X‐ray detectors.

[CCDC 238405 and 2384420 contain the supplementary crystallographic data for this paper. These data can be obtained free of charge from The Cambridge Crystallographic Data Centre via www.ccdc.cam.ac.uk/data_request/cif.]

## Conflict of Interest

The authors declare no conflict of interest.

## Supporting information



Supporting Information

Supporting cif

## Data Availability

The data that support the findings of this study are available in the supporting information of this article.
