# Supplementary material for: Multiple Interactions in Polar Lead‐Free Perovskites toward Highly Stable X‐Ray Detection
Source: Adv Sci (Weinh). 2025 Apr 3;12(20):2412504. doi: 10.1002/advs.202412504 (PMC12120703; doi:10.1002/advs.202412504)
Supplement: Supplementary file 1 — Supporting Information [file ADVS-12-2412504-s002.docx]

#### Supporting Information

**Multiple Interactions in Polar Lead-Free Perovskites towards Highly Stable X-ray Detection**

*Chang Qu, Jianbo Wu, Zeng-kui Zhu, Qianwen Guan, Huang Ye, Ruiqing Li, Chengshu Zhang, Yaru Geng, Hang Li, Lijun Xu, Haiqing Zhong, Ailin Wang, Chengmin Ji, Zhenyue Wu* and* *Junhua Luo****

**Experimental Details**

**Chemicals: (***R*)-(+)-1-(4-Bromophenyl)ethylamine (*R*-BPEA, Aladdin, 98%), (*S*)-(-)-1-(4-Bromophenyl)ethylamine (*S*-BPEA, Aladdin, 98%), **(***R*)-(+)-ɑ-Methylbenzylamine (*R*-PEA, Aladdin, 99%), **(***S*)-(-)-1-Phenylethylamine (*S*-PEA, Aladdin, 99%), bismuth oxide (Bi_2_O_3_, 99%, Aladdin), hydroiodic acid solution (HI, 55%∼58%, Aladdin), barium sulfate (BaSO_4_, AR, Aladdin). All the chemicals were bought and used without further purification.

**Synthesis and crystals growth:** Crystalline materials of (*R*/*S*-BPEA)_2_BiI_5_ (**1*R***/**1*S***) were synthesized by dissolving Bi_2_O_3_ (2 mmol; 930 mg) and *R*-BPEA (4 mmol; 800 mg) or *S*-BPEA (4 mmol; 800 mg) in HI solution (20 mL) by heating to boiling under a constant magnetic stirring. After a clear red solution was obtained, the heating and stirring was stopped. The crystallization temperature should be controlled near 50 ℃, then the high-quality single crystals with large dimension can be grown from its saturated solution via a temperature-cooling approach in an oven with a slow decrease rate of 1K day^−1^. The same method was used to grow (*R*/*S*-PEA)_2_BiI_5_ (**2*R***/**2*S***) high-quality single crystals.

**Single Crystal X-ray Diffraction:** The diffraction data were collected by using graphite-monochromatized Cu Kα radiation (λ= 1.5418 Å) at 293(2) K on an Agilent SuperNova Dual

diffractometer with an Atlas detector. The collection of the intensity data, cell refinement, and

data reduction were carried out with the program CrysAlisPro. The crystal structure was solved by the direct method and refined by the full-matrix method based on F2 using the SHELXTL

program. CCDC 2384205 and 2384420 contains the supplementary crystallographic data for compound **1*R*/1*S***.

**Material characterization:** Powder XRD patterns were measured on a Rigaku Miniflex 600 X-ray diffractometer in the 2𝜃 range of 5°–50° with a step width of 0.02°. The surface micromorphology and corresponding elemental distribution mappings of single crystals were acquired using a field emission electron microscope (JSM6700-F, JEOL) attached to an energy-dispersive Xray spectroscope (X-Max^N^, Oxford Instrument). The absorption spectrum was recorded on a UV−vis-NIR spectrometer (Lambda 950, PerkinElmer). Thermogravimetric curves were measured on a STA F4 Jupiter simultaneous thermal analyzer (Netzsch) in a N_2_ atmosphere from room temperature to 800 °C with a heating speed of 10 °C min^−1^. The temperature-dependent conductivities of single crystals were derived from their resistance−temperature curves, which were measured using a high-precision electrometer (6517B, Keithley) by placing samples on a heating−cooling stage (THMS600, Linkam).

**X-Ray Detection:** The *I*–*V* traces and *I*–*t* curves of the **1*S*** device were recorded by a Keithley 6517B electrometer under X-ray irradiation. An Amptek Mini-X2 X-ray tube with an Ag target (maximum power 4 W) was used as the X-ray source. The maximum X-ray photons energy was 50 keV and the peak intensity was at 22 keV. The dose rate of X-ray tube can be modulated by changing its tube current and measured by a Radcal Accu-Gold X-ray dosimeter attached with the 10×6-180 ion chamber in an integrating mode.

**Figures and Tables**

**Table S1.** Crystal data and structure refinements for **1*R*/1*S*** at 300 K.

|  | (*R*-BPEA)_4_Bi_2_I_10_ (**1*R***) | (*S*-BPEA)_4_Bi_2_I_10_ (**1*S***) |
| --- | --- | --- |
| Formula | (C_8_H_11_NBr)_4_Bi_2_I_10_ | (C_8_H_11_NBr)_4_Bi_2_I_10_ |
| Weight | 2491.31 | 2491.31 |
| Temperature [K] | 300 | 300 |
| Crystal system | monoclinic | monoclinic |
| Space group | *P*2_1_ | *P*2_1_ |
| a [Å] | 13.36530(10) | 13.3645(3) |
| b [Å] | 14.30600(10) | 14.3035(3) |
| c [Å] | 31.1689(3) | 31.1685(6) |
| α [°] | 90 | 90 |
| β [°] | 94.7060(10) | 94.690(2) |
| γ [°] | 90 | 90 |
| Volume [Å^3^] | 5939.53(8) | 5938.2(2) |
| *Z* | 4 | 4 |
| *ρ*_calc._ [g/cm^3^] | 2.786 | 2.787 |
| μ [mm^‑1^] | 55.654 | 55.667 |
| F(000) | 4400.0 | 4400.0 |
| Radiation | Cu Kα (*λ* = 1.54184 Å) | Cu Kα (*λ* = 1.54184 Å) |
| 2Ɵ range for data collection [°] | 5.69 ~ 153.026 | 5.69 ~ 134.998 |
| Index ranges | -16 ≤ *h* ≤ 16,  -14 ≤ *k* ≤ 17  -38 ≤ *l* ≤ 39 | -16 ≤ *h* ≤ 16  -17 ≤ *k* ≤ 13  -37 ≤ *l* ≤ 37 |
| Reflections collected | 43722 | 42034 |
| Independent reflections | 17854 [*R*_int_ = 0.0761, *R*_sigma_ = 0.0653] | 16866 [*R*_int_ = 0.0890, *R*_sigma_ = 0.0958] |
| Final *R* indexes [*I* ≥ 2*σ* (*I*)] | *R*_1_ = 0.0653, *wR*_2_ = 0.1648 | *R*_1_ = 0.0818, *wR*_2_ = 0.2150 |
| Final *R* indexes [all data] | *R*_1_ = 0.0690, *wR*_2_ = 0.1690 | *R*_1_ = 0.0965, *wR*_2_ = 0.2292 |
| GOF | 1.005 | 1.066 |

**
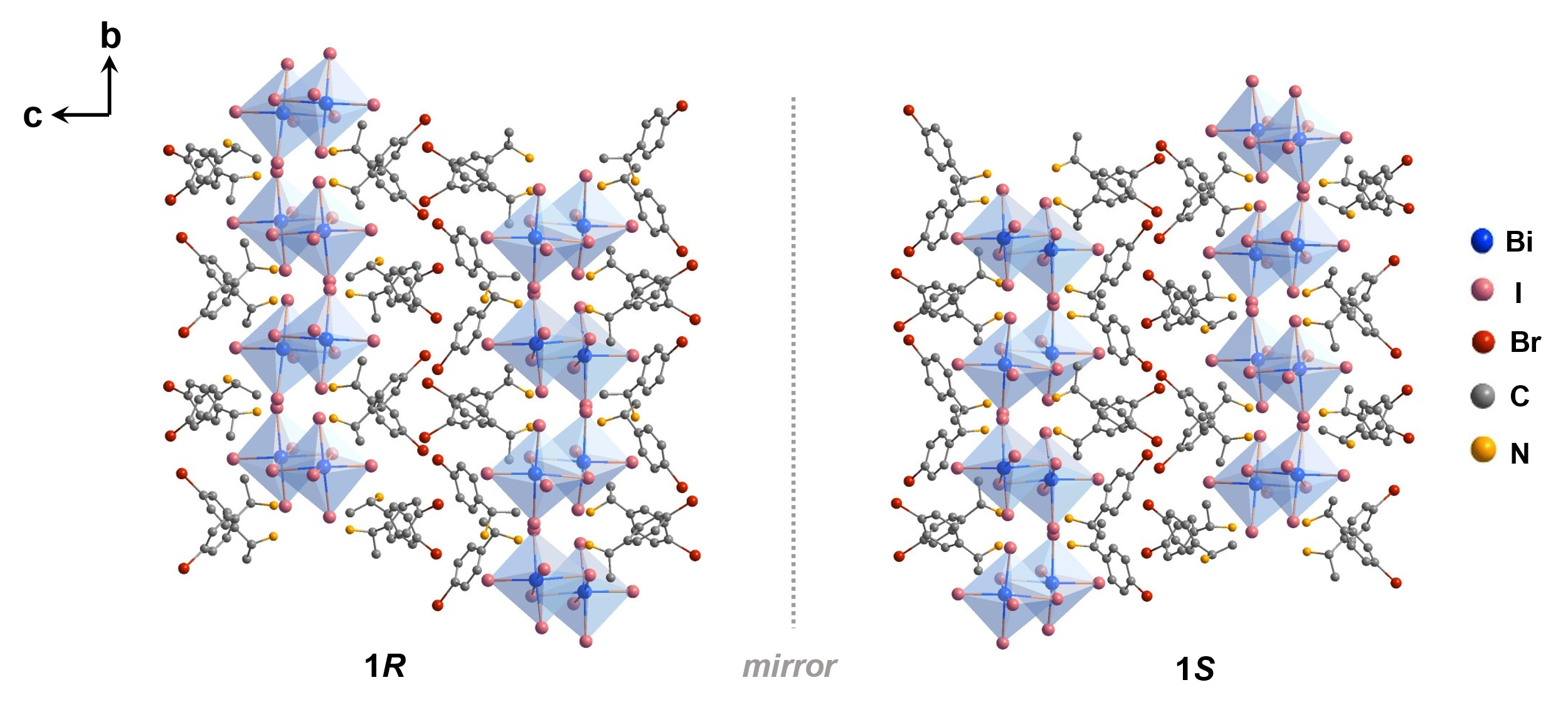
**

**Figure S1.** Enantiomerically packed structures of **1*R*** and **1*S*** are viewed along the *a*-axis. Hydrogen atoms have been omitted.


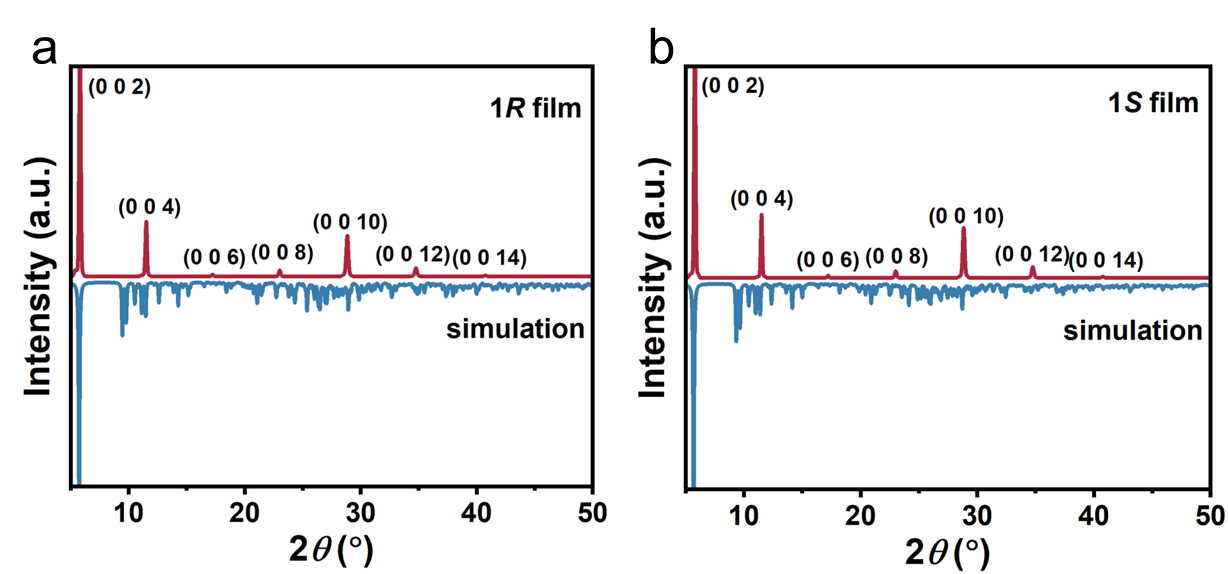


**Figure S2.** The XRD patterns of **1*R*** and **1*S*** films in comparison to their simulated patterns.


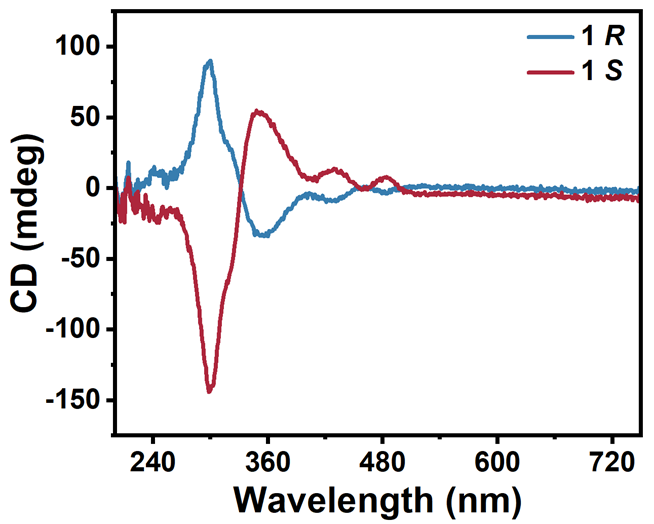


**Figure S3.** CD spectra for **1*R*** and **1*S***.

**
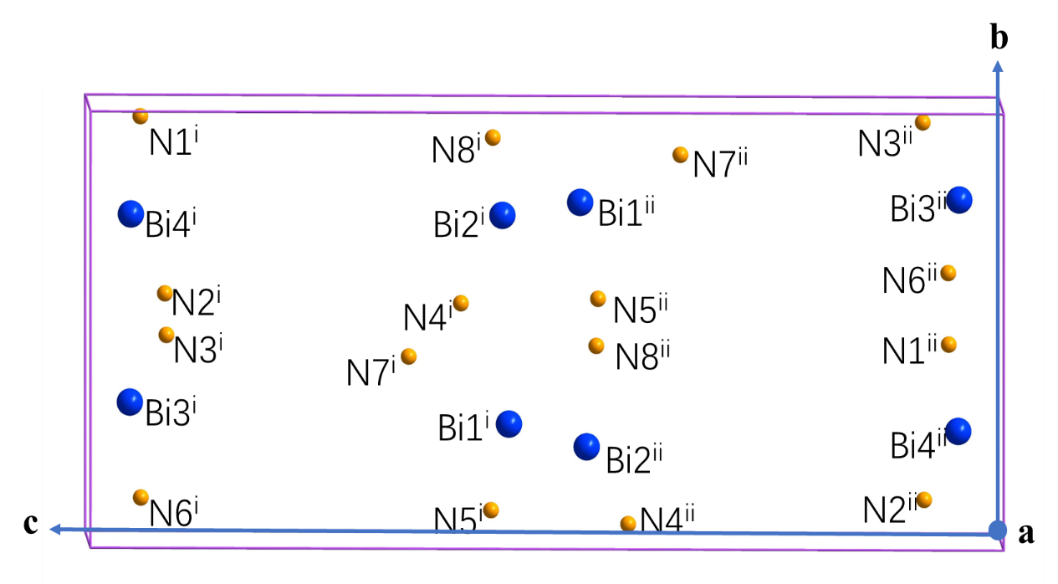
**

**F****igure S4.** Distribution of Bi and N atoms in a unit cell of **1*S***.

The spontaneous electric polarization intensity (*P*_s_) calculation is based on the crystal structure data of **1*S*** collected at 300 K. We select a unit cell and assume that the centers of the positive charges of *S*-BPEA^+^ and the negative charges of the [Bi_2_I_10_]^4-^ are located on the N and Bi atoms, respectively.

**Table S2.** Atomic coordinates for Bi and N in **1*S***.

| Atoms | Atomic coordinate | | Coordinate of charge center |
| --- | --- | --- | --- |
| Bi | Bi1^i^(0.61060, 0.27110, 0.53960) | Bi1^ii^(0.38940, 0.77110, 0.46040) | (0.5, 0.5125, 0.5) |
|  | Bi2^i^(0.11530, 0.73030, 0.54390) | Bi2^ii^(0.88470, 0.23030, 0.45610) |  |
|  | Bi3^i^(0.10470, 0.29910, 0.95170) | Bi3^ii^(0.89530, 0.79910, 0.04830) |  |
|  | Bi4^i^(0.60280, 0.74950, 0.95360) | Bi4^ii^(0.39720, 0.24950, 0.04640) |  |
| N | N1^i^(0.27500, 0.96100, 0.94130) | N1^ii^(0.72500, 0.46100, 0.05870) | (0.5, 0.50663, 0.5) |
|  | N2^i^(0.91500, 0.58000, 0.91820) | N2^ii^(0.08500, 0.08000, 0.08180) |  |
|  | N3^i^(0.40700, 0.46500, 0.91350) | N3^ii^(0.59300, 0.96500, 0.08650) |  |
|  | N4^i^(0.43600, 0.54100, 0.59120) | N4^ii^(0.56400, 0.04100, 0.40880) |  |
|  | N5^i^(0.31000, 0.06200, 0.55740) | N5^ii^(0.69000, 0.56200, 0.44260) |  |
|  | N6^i^(0.76400, 0.10600, 0.94360) | N6^ii^(0.23600, 0.60600, 0.05640) |  |
|  | N7^i^(0.04000, 0.40300, 0.64600) | N7^ii^(0.96000, 0.90300, 0.35400) |  |
|  | N8^i^(0.80300, 0.93500, 0.55850) | N8^ii^(0.19700, 0.43500, 0.44150) |  |

*P*_s_ =(q_N_r_N_ + q_Bi_r_Bi_) × Z × b / V

=[(e×0.50663)×16 + (-3e×0.5125)×8×2/3]×b/V

=(0.50663×16-0.5125×16)×1.6×10-19×14.3035×10-10/(5938.2×10-30) C m^-2^

=-0.00587×16×1.6×14.3035/5938.2×10 C m^-2^

≈-0.00362 C m^-2^

|*P*_s_|≈0.00362 C m^-2^≈0.362 μC cm^-2^

**
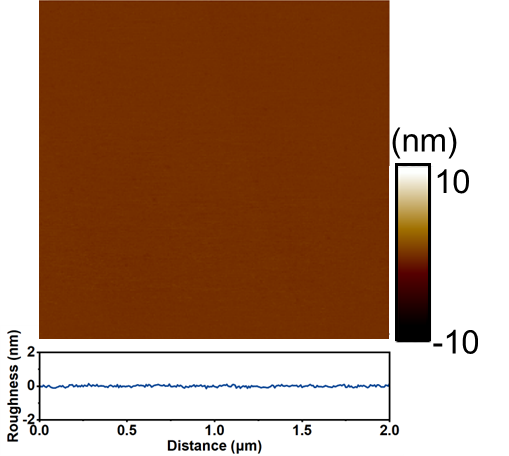
**

**Figure S5.** AFM image of **1*S*** (00*l*) crystal surface.

**
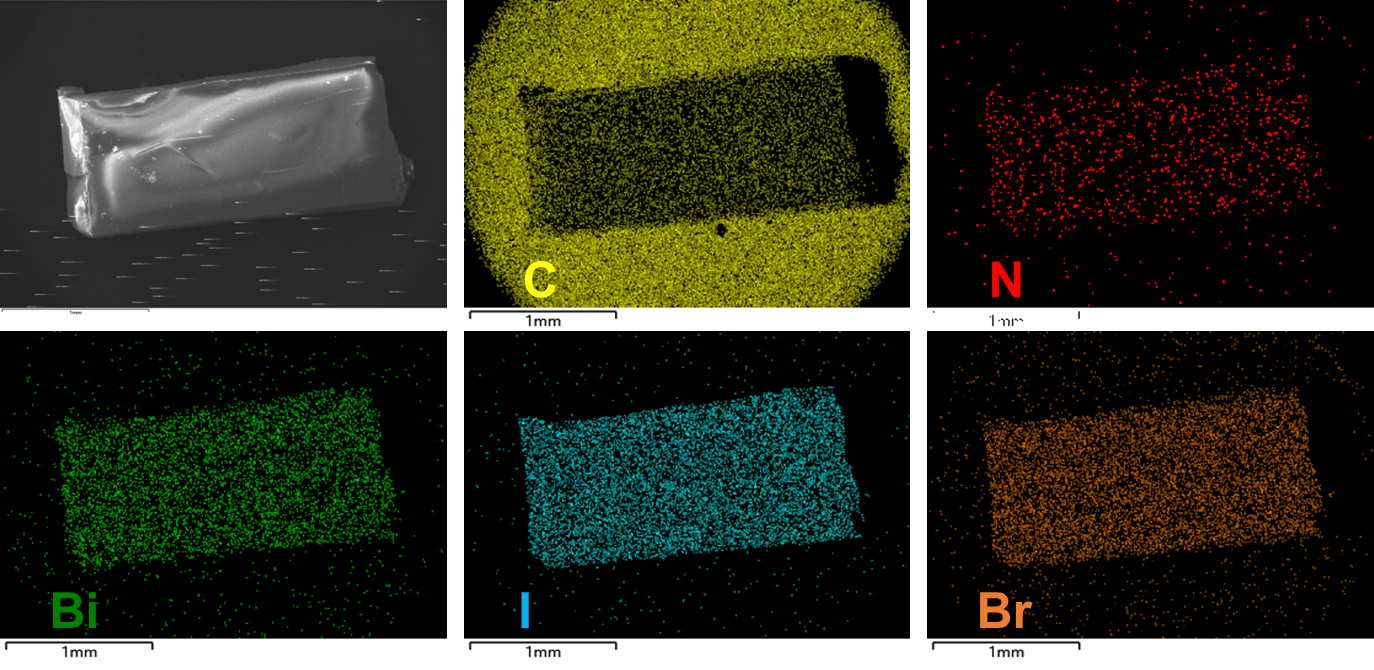
**

**Figure S6.** SEM image and corresponding EDS mappings of the surface of **1*S*** SC.

**
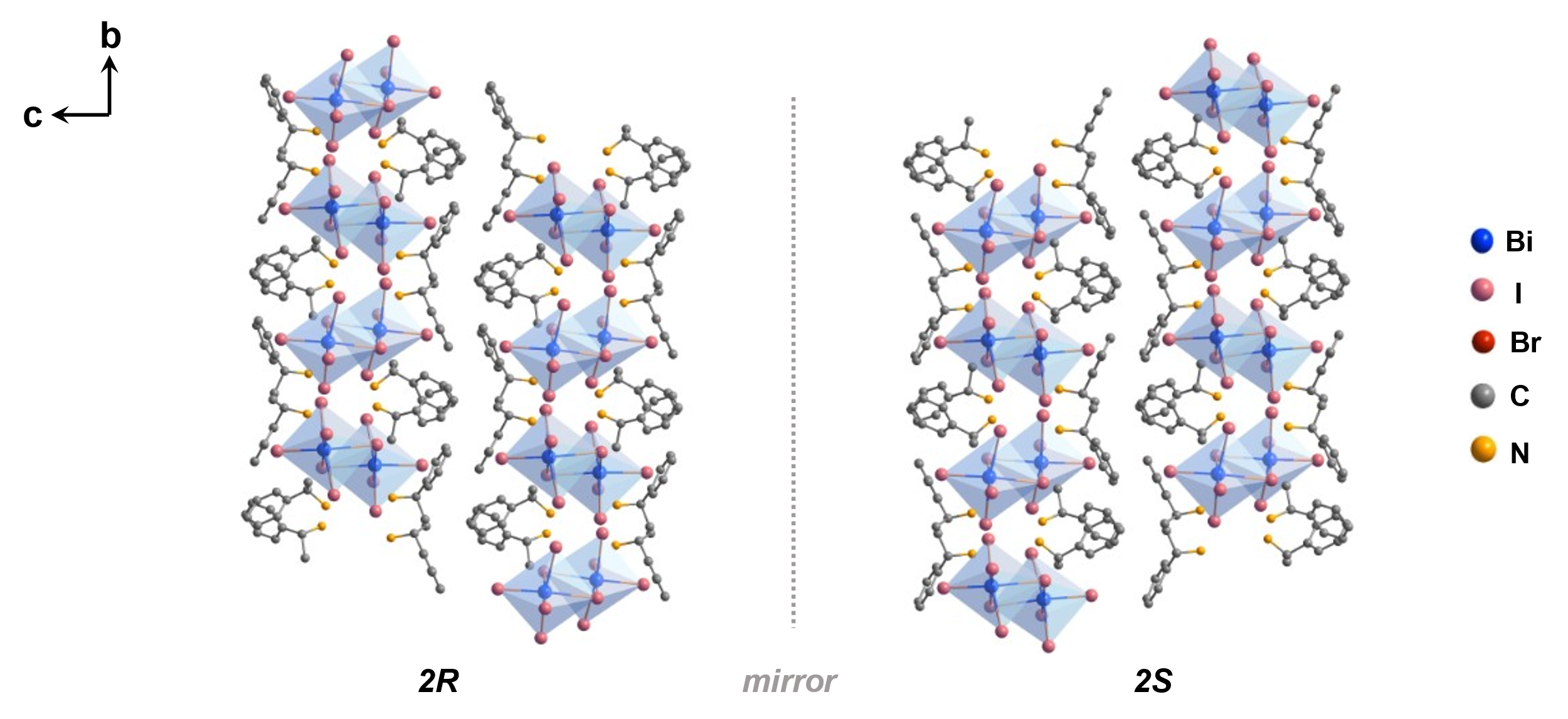
**

**Figure S7.** Enantiomerically packed structures of **2*R*** and **2*S*** are viewed along the *a*-axis. Hydrogen atoms have been omitted.

**
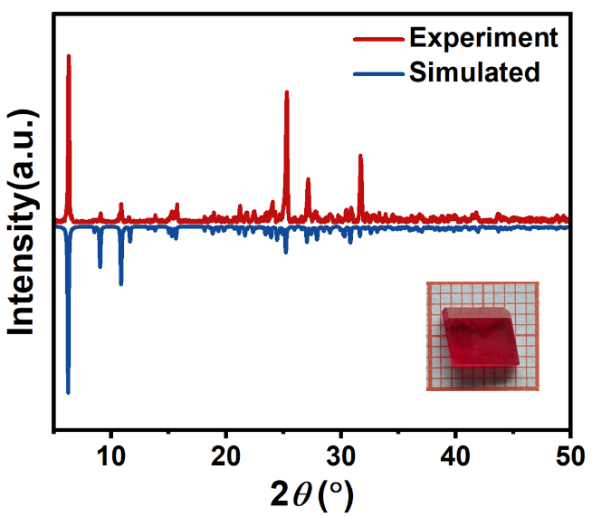
**

**Figure S8.** The experimental and simulated powder XRD patterns of **2*S***. The inset is the photographs of bulk crystals.


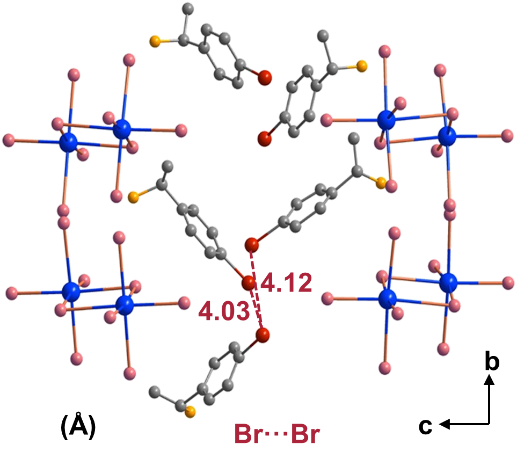


**Figure S9.** Distances from Br atoms to neighboring Br atoms in the structure of **1*S***.

**
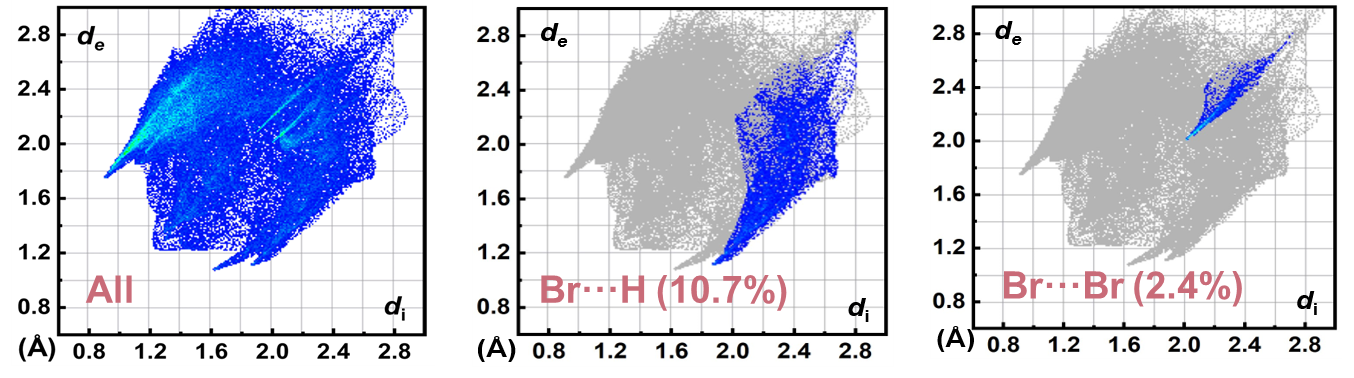
**

**Figure S10.** Hirshfeld surface analyses of the BPEA cations and their corresponding 2D fingerprints of all, Br∙∙∙H, Br∙∙∙Br.

**

**

**Figure S11.** TG curves of **1*S*** and **2*S***, respectively.

**
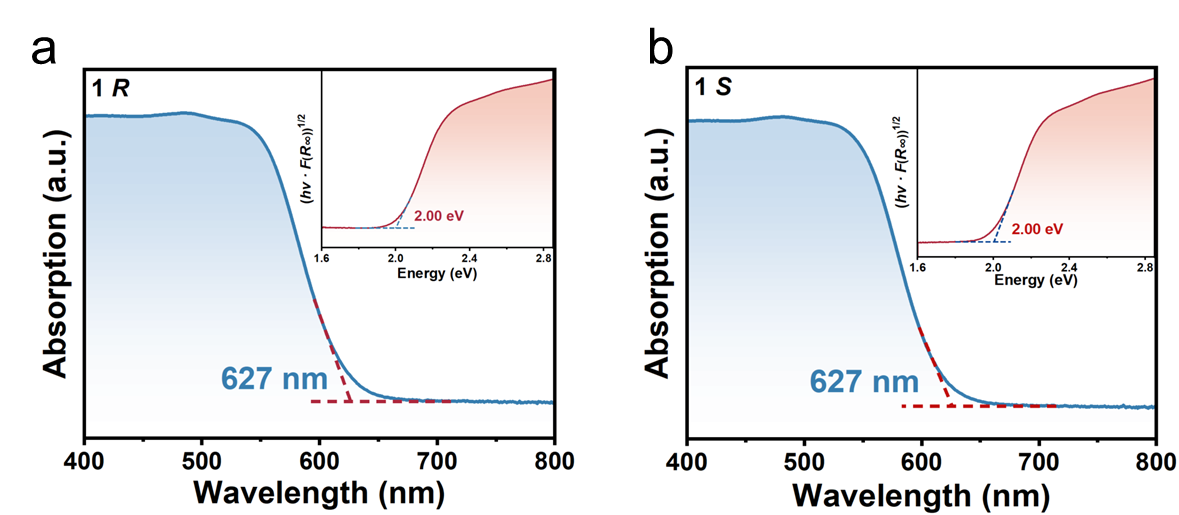
F****igure S12.** The absorption spectra of **1*R*** and **1*S***. The inset shows the experimental bandgap.


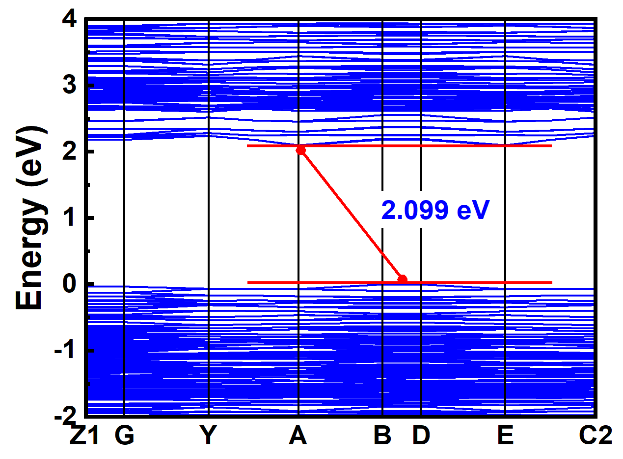


**Figure S13.** The calculated bandgap of **1*S***.

^
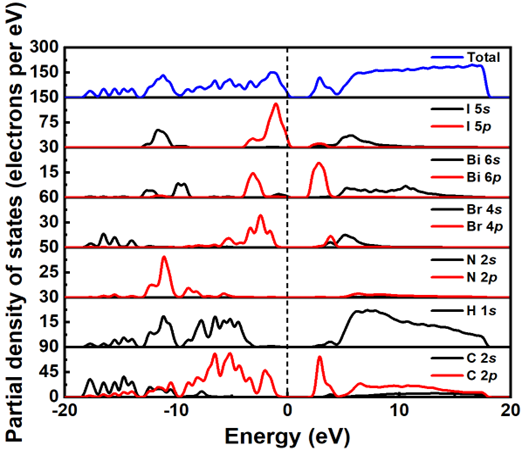
^

**Figure S14.** Partial density of states of **1*S***.

**
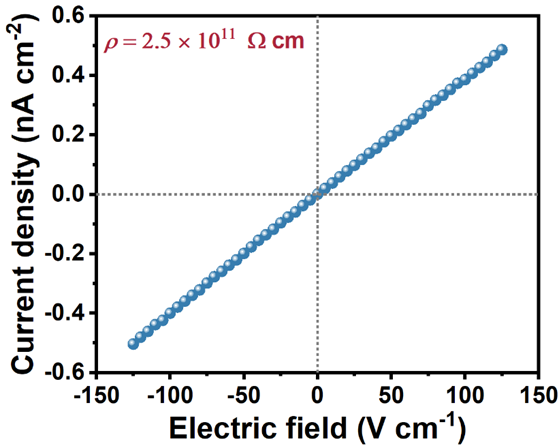
**

**Figure S15.** Bulk resistivity of **1*S*** SC.


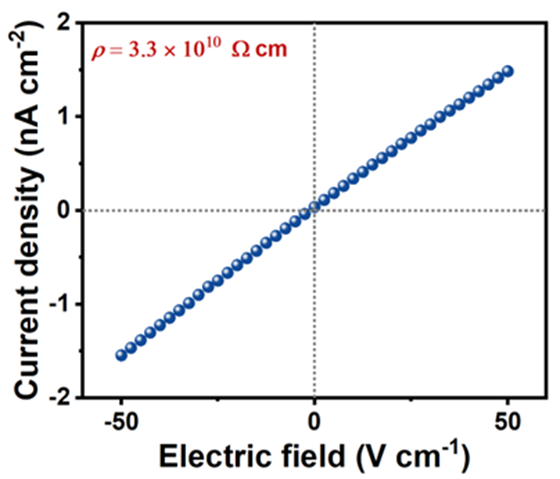


**Figure S16.** Bulk resistivity of **2*S*** SC.


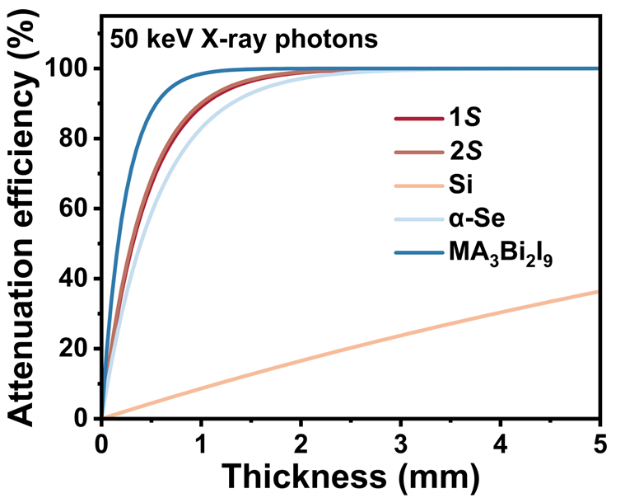


**Figure S17.** Attenuation efficiencies of **1*S***, **2*S***, Si, α-Se, MA_3_Bi_2_I_9_ for 50-keV X-ray photons versus thickness.


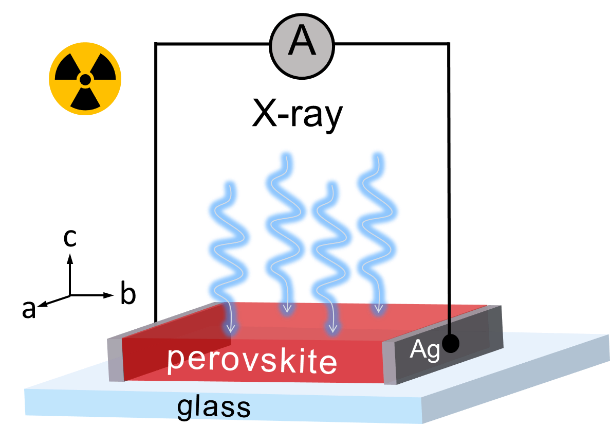


**Figure S18.** Schematic illustration of **1*S*** SC detector.


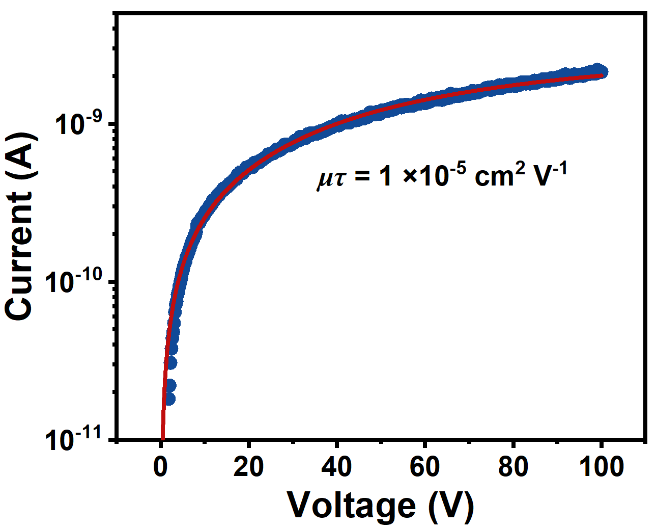
**Figure S19.** Voltage-dependent photoconductivity of the **2*S*** SC device.


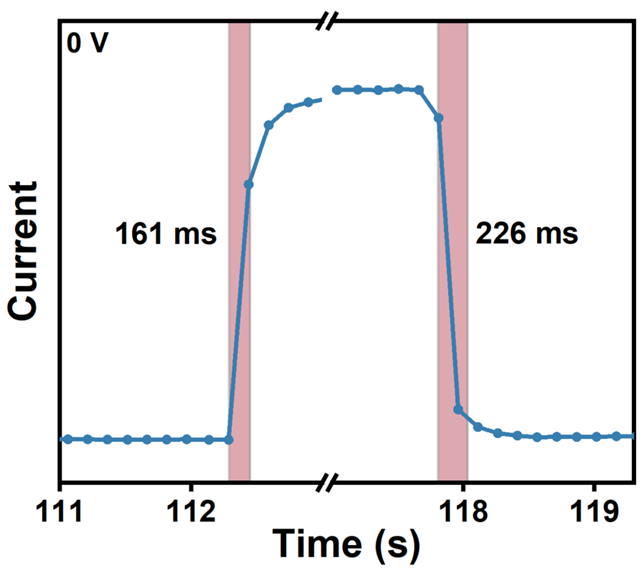


**Figure S20.** Response time of **1*S*** SC detector under X-ray switching.


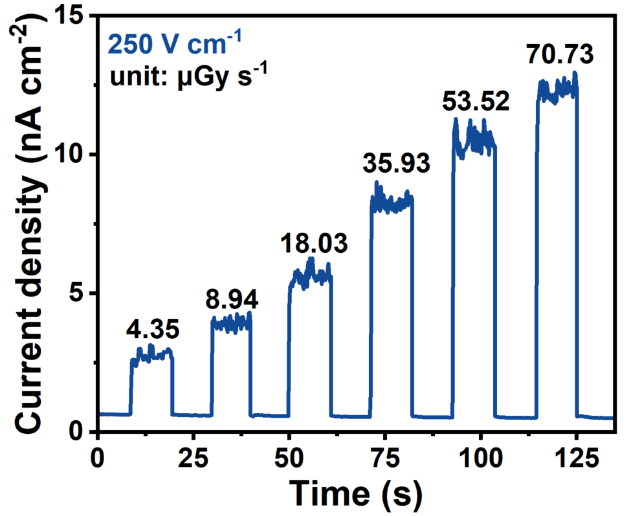


**Figure S21.** *J-t* curves of **1*S*** SC detector under increased X-ray dose rates at 250 V cm^-1^.


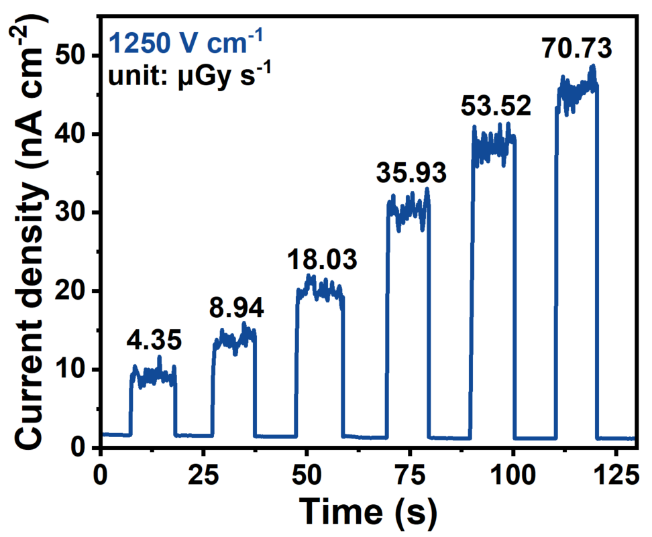


**Figure S22.** *J-t* curves of **1*S*** SC detector under increased X-ray dose rates at 1250 V cm^-1^.


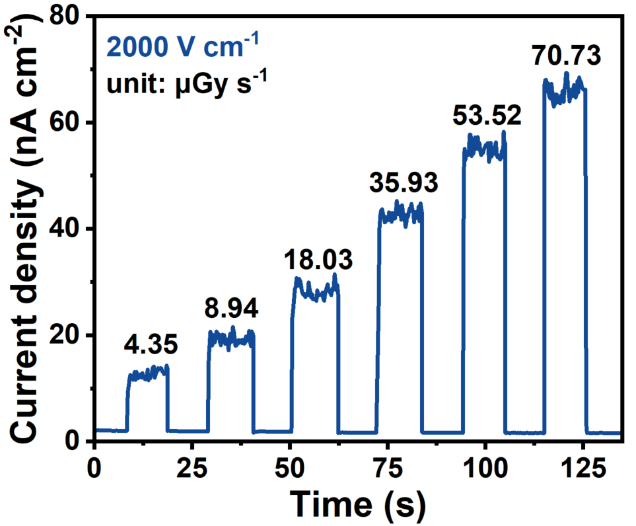


**Figure S23.** *J-t* curves of **1*S*** SC detector under increased X-ray dose rates at 2000 V cm^-1^.


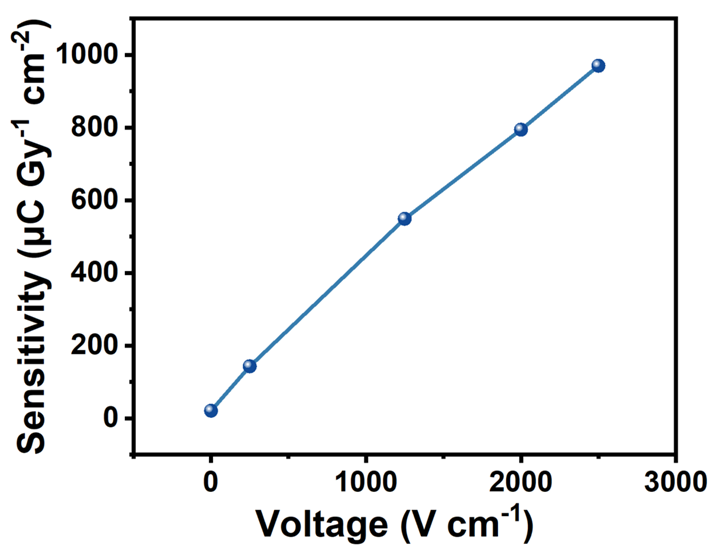


**Figure S24.** The relationship between sensitivity and external electric field.

**
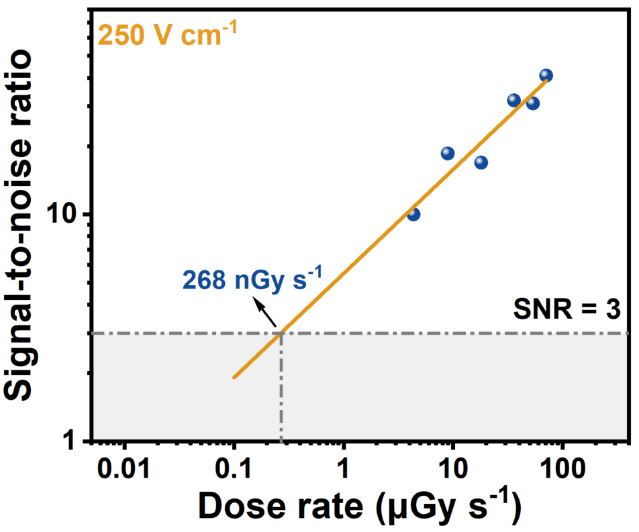
Figure S25.** SNRs of **1*S*** detector under different dose rates at 250 V cm^-1^, which derives the LoD of 268 nGy s^-1^.


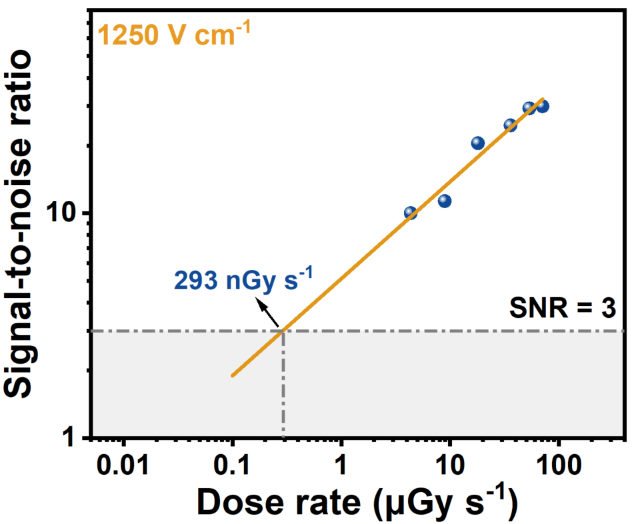


**Figure S26.** SNRs of **1*S*** detector under different dose rates at 1250 V cm^-1^, which derives the LoD of 293 nGy s^-1^.


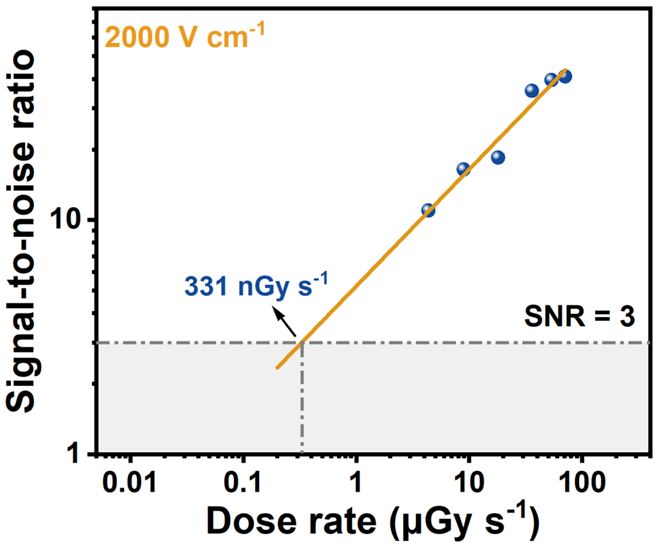


**Figure S27.** SNRs of **1*S*** detector under different dose rates at 2000 V cm^-1^, which derives the LoD of 331 nGy s^-1^.


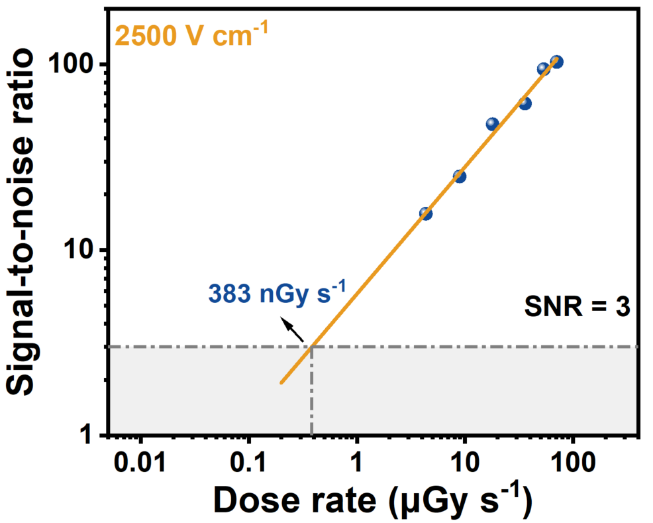


**Figure S28.** SNRs of **1*S*** detector under different dose rates at 2500 V cm^-1^, which derives the LoD of 358 nGy s^-1^.


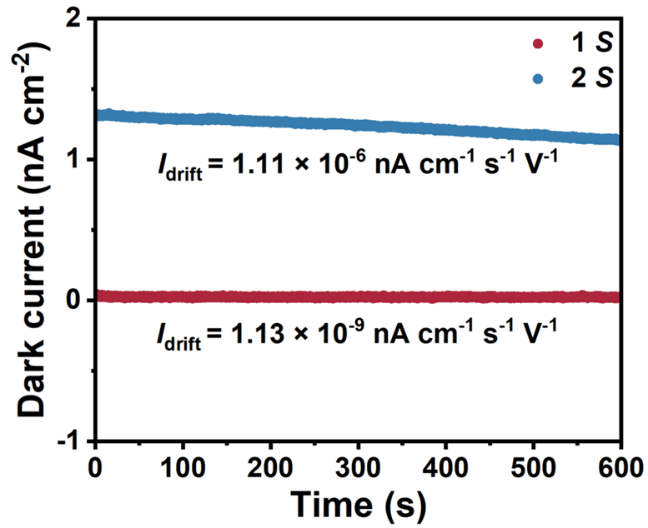


**Figure S29.** Dark current tracking of **1*S*** and **2*S*** detector at 250 V cm^-1^.

**Table S3.** Performances of some reported X-ray detectors.

| **Compound** | **Dimensionality** | ***μτ***  **(cm^2^ V^-1^)** | **Electric filed**  **(V cm^-1^)** | ***S***  **(μC Gy^-1^ cm^-2^)** | ***LoD***  **(nGy s^-1^)** | ***I*_drift_**  **(nA cm^-1^ s^-1^ V^-1^)** | **Refs** |
| --- | --- | --- | --- | --- | --- | --- | --- |
| **(S-BPEA)_4_Bi_2_I_10_** | **0 D** | **1.4 × 10^-5^** | **Self-driven** | **21** | **183** | **3.25×10^-8^(100 V)** | **This work** |
|  |  |  | **100 V** | **970** | **383** |  |  |
| (R-PPA)_2_BiI_5_ | 0 D | 5.6 × 10^-5^ | Self-driven | 31 | 316 | / | [1] |
|  |  |  | 10 V | 150 | 2570 | 1.0×10^-4^ |  |
| FA_3_Bi_2_I_9_ | 0 D | 2.4 × 10^-5^ | 2000 | 598.1 | 200 | / | [2] |
| (CH_3_NH_3_)_3_Bi_2_I_9_ | 0 D | 2.87 × 10^-3^ | 600 | 1947 | 83 | 5.0×10^-10^(10 V) | [3] |
| Cs_3_Bi_2_I_9_ | 0 D | 7.97 × 10^-4^ | 500 | 1652.3 | 130 | / | [4] |
| Gua_3_Bi_2_I_9_ | 0 D | / | 500 V | 18.23 | 237.54 | / | [5] |
| AG_3_Bi_2_I_9_ | 0 D | 7.94 × 10^-3^ | 10 | 5791 | 2.6 | 7.3 × 10^-8^(200 V cm^-1^) | [6] |
| (BZA)_2_(R/S-PPA)BiI_6_ | 0 D | 3.08 × 10^-5^ | Self-driven | 53.2 | 18.5 | / | [7] |
|  |  |  | 50 V | 2170 | / |  |  |
| (HIS)BiI_5_ | 0 D | 2.81 × 10^−4^(010) | 50 V | 1230 | 36.4 | 3.345×10^-6^(10 V) | [8] |
|  |  | 0.85 × 10^−4^(001) |  | 1133.1 |  | 2.559×10^-7^(10 V) |  |
| (BAH)BiI_4_ | 1 D | 1.95 × 10^−4^ | 50 V | 1181.8±32.3 | 77 | / | [9] |
| (4-AMP)BiI_5_ | 1 D | 2.35 × 10^−4^ | Self-driven | 66.84 | 482 | 1.51 × 10^−5^ (50 V) | [10] |
| (NH_4_)_3_Bi_2_I_9_ | 2 D | 1.1 × 10^-2^  (parallel） | 22 | 8000 | 210 | / | [11] |
|  |  | 4.0 × 10^-3^  (perpendicular） | 65 | 803 | 55 | / |  |
| Rb_3_Bi_2_I_9_ | 2 D | 2.51 × 10^-3^ | 10 | 42.5 | 8.32 | 1.82 × 10^-7^（100 V） | [12] |
| (R-MPA)_4_AgBiI_8_ | 2 D | 2.2 × 10^−5^ | Self-driven | 46.3 | 85 | / | [13] |
|  |  |  | 50 V | 949.6 | 547 | 1.02×10^-3^ |  |
| [(R/S-PPA)_4_(IPA)_6_Ag_2_Bi_4_I_24_]·  2H_2_O | 2 D | 2.44 × 10^-4^ | 30 | 42.8 | 129.5 | / | [14] |
| (4-AP)_2_AgBiBr_8_ | 2 D | 4.8 × 10^−4^ | 80 V | 1117.3 | 810 | 3.06 × 10^−8^ | [15] |
| (DFPIP)_4_AgBiI_8_ | 2 D | 1 × 10^-5^ | 50 V | 188 | 3130 | / | [16] |
| (FPEA)_4_AgBiBr_8_ | 2 D | 2.9 × 10^-5^ | 33.3 | 27 | 2600 | / | [17] |
| (PEA)_4_AgInBr_8_ | 2 D | 2.0 × 10^-3^ | 20 | 185 | / | 7.33 × 10^-7^(20 V mm^-1^) | [18] |
| (4,4-DFPD)_4_AgSbI_8_ | 2 D | 6.19 × 10^-4^ | 100 V | 704.8 | 360 (10 V) | / | [19] |
| (BA)_2_CsAgBiBr_7_/Cs_2_AgBiBr_6_ | 2 D/3 D | 2.8 × 10^−3^ | Self-driven | 206 | / | / | [20] |
| Cs_2_AgBiBr_6_ | 3 D | 3.75 × 10^−3^  (pristine) | 50 V | 105 | 59.7 | / | [21] |
|  |  | 6.3 × 10^−3^  (Annealed) |  |  |  |  |  |
|  |  | 6.0 × 10^−3^  (Annealed + surface treatment) |  |  |  |  |  |
| ɑ-Se film | / | 10^-7^ | 100 | 20 | 5500 | / | [22] |

**References**

1. S. You, Z.-K. Zhu, S. Dai, J. Wu, Q. Guan, T. Zhu, P. Yu, C. Chen, Q. Chen, J. Luo, *Adv. Funct. Mater.* **2023**, 33, 2303523.
2. W. Li, D. Xin, S. Tie, J. Ren, S. Dong, L. Lei, X. Zheng, Y. Zhao, W.-H. Zhang, *J. Phys. Chem. Lett.* **2021**, 12, 1778.
3. Y. Liu, Z. Xu, Z. Yang, Y. Zhang, J. Cui, Y. He, H. Ye, K. Zhao, H. Sun, R. Lu, M. G. Kanatzidis, S. Liu, *Matter* **2020**, 3, 180.
4. Y. Zhang, Y. Liu, Z. Xu, H. Ye, Z. Yang, J. You, M. Liu, Y. He, M. G. Kanatzidis, S. F. Liu, *Nat. Commun.* **2020**, 11, 2304.
5. Y. Xu, J. Hu, X. Xiao, H. He, G. Tong, J. Chen, Y. He, *Inorg. Chem. Front.* **2022**, 9, 494.
6. M. Chen, X. Dong, D. Chu, B. Jia, X. Zhang, Z. Zhao, J. Hao, Y. Zhang, J. Feng, X. Ren, Y. Liang, R. Shi, A. Najar, Y. Liu, S. Liu, *Adv. Mater.* **2023**, 35, 2211977.
7. Z.-K. Zhu, T. Zhu, S. You, P. Yu, J. Wu, Y. Zeng, Q. Guan, Z. Li, C. Qu, H. Zhong, L. Li, J. Luo, *Small* **2023**, 2307454.
8. Z. Zhao, Q. Fan, Y. Liu, H. Rong, H. Ni, L. Wei, X. Zhao, J. Luo, Z. Sun, *ACS Appl. Mater. Interfaces* **2024**, 16, 38283.
9. C. Ma, D. Shen, T.-W. Ng, M.-F. Lo, C.-S. Lee, *Adv. Mater.* **2018**, 30, 1800710.
10. S. You, P. Yu, T. Zhu, C. Lin, J. Wu, Z.-K. Zhu, C. Zhang, Z. Li, C. Ji, J. Luo, *Adv. Funct. Mater.* **2023**, 2310916.
11. R. Zhuang, X. Wang, W. Ma, Y. Wu, X. Chen, L. Tang, H. Zhu, J. Liu, L. Wu, W. Zhou, X. Liu, Y. Yang, *Nat. Photo.* **2019**, 13, 602.
12. M. Xia, J.-H. Yuan, G. Niu, X. Du, L. Yin, W. Pan, J. Luo, Z. Li, H. Zhao, K.-H. Xue, X. Miao, J. Tang, *Adv. Funct. Mater.* **2020**, 30, 1910648.
13. J. Wu, S. You, P. Yu, Q. Guan, Z.-K. Zhu, Z. Li, C. Qu, H. Zhong, L. Li, J. Luo, *ACS Energy Lett.* **2023**, 8, 2809.
14. Z.-K. Zhu, T. Zhu, J. Wu, S. You, P. Yu, X. Liu, L. Li, C. Ji, J. Luo, *Adv. Funct. Mater.* **2023**, 33, 2214660.
15. G. Chen, H. Dai, Z.-K. Zhu, J. Wu, P. Yu, Y. Zeng, Y. Zheng, L. Xu, J. Luo, *Small* **2024**, 2312281.
16. C.-F. Wang, H. Li, M.-G. Li, Y. Cui, X. Song, Q.-W. Wang, J.-Y. Jiang, M.-M. Hua, Q. Xu, K. Zhao, H.-Y. Ye, Y. Zhang, *Adv. Funct. Mater.* **2021**, 31, 2009457.
17. M. Ge, S. Chen, X. Fu, Y. Feng, D. Wang, M. Yuan, *J. Phys. Chem. C* **2022**, 126, 19417.
18. D. Chen, G. Niu, S. Hao, L. Fan, J. Zhao, C. Wolverton, M. Xia, Q. Liu, *ACS Appl. Mater. Interfaces* **2021**, 13, 61447.
19. C. F. Wang, H. Li, M. G. Li, Y. Cui, X. Song, Q. W. Wang, J. Y. Jiang, M. M. Hua, Q. Xu, K. Zhao, H. Y. Ye, Y. Zhang, *Adv. Funct. Mater.* **2021**, 31, 2009457.
20. X. Zhang, T. Zhu, C. Ji, Y. Yao, J. Luo, *J. Am. Chem. Soc.* **2021**, 143, 20802.
21. W. Pan, H. Wu, J. Luo, Z. Deng, C. Ge, C. Chen, X. Jiang, W.-J. Yin, G. Niu, L. Zhu, L. Yin, Y. Zhou, Q. Xie, X. Ke, M. Sui, J. Tang, *Nat. Photon.* **2017**, 11, 726.
22. H. Wei, J. Huang, *Nat. Commun.* **2019**, 10, 1066.
